# Medical Opinion and Movement

**Published:** 1908-10-31

**Authors:** 


### Medical Opinion and Movement.

In the current number of the *Journal of the Royal Army Medical Corps* appears a short article by Colonel Hathaway and Major Blackham on the "Red Cross of Geneva," which is deserving of a wider reading public than it is likely to obtain in the journal. The article is the first chapter of a short handbook on "Ambulance Transport," on which the authors are at present engaged. Such a work, we are sure, will be welcomed by many, and there is need for a short and concise history of the evolution of medical service in war time. The authors trace the gradual development of the neutrality of hospitals and nursing *personnel*, and, we are glad to see, give due credit to Signor Polaschiano, while not forgetting the meed of praise to M. Dunant. The article raises certain questions which it is very tempting to discuss—questions such as the position of the Red Cross Societies and the Order of St. John, and the responsibility of maintaining an effective army ambulance service in peace time. It gives a full and accurate account of the rise and development of red cross work in England, and recognises fully the valuable services that volunteer help committees have rendered in the past as auxiliaries to the official corps.

In the *Boston Medical and Surgical Journal*, J. Friedenwald discusses a condition of the stomach known as gastro-myxorrhœa. According to Kuttner the normal fasting stomach should never contain more than 5 c.c. of mucus. If mucus be present in quantity greater than 25 c.c. he terms the condition one of gastro-myxorrhœa. The presence of mucus may be constant or intermittent. The latter form is rare, and is accompanied by cephalalgia, loss of appetite, nausea, etc. The patient vomits large quantities of liquid, and later of bile. The abdomen is retracted, the tongue dry, and the pulse small. An attack lasts from five to ten days. On examination the gastric juice is found to contain the normal percentage of hydrochloric acid, but an excess of mucus is present. The usual treatment consists in washing out the stomach at the commencement of an attack. The author, however, has no faith in this treatment, except as a palliative to relieve symptoms. Temporary relief can be procured by morphia. Lavages of saline fluid, accompanied by subcutaneous injections of artificial serum, are useful in cases in which there is much prostration. Change of climate, hydrotherapy, and massage are indicated when the patient is of nervous temperament. In some cases a rest cure has had good effect. The most useful drugs are iron and arsenic. The chronic or constant form is much the more common variety met with. Its pathology is rather uncertain, some authorities considering it a neurosis and others that it is associated with a chronic catarrhal condition of the stomach. Farinaceous food would seem to predispose to the condition. There are no characteristic symptoms of the disease, diagnosis being confirmed by the presence of large quantities of mucus in the fasting stomach after elimination of other pathological conditions such as nasal and pharyngeal disease from which quantities of mucus might find their way into the stomach. In the matter of treatment, although gastric lavage is quite allowable when no contradistinction to its use is found, the author prefers to use mineral waters, which he chooses to suit the particular nature of the case under treatment.

Dr. A. Borri, of Parma, has made some interesting observations on the toxicity of the fluid from gastric lavage under varying conditions. The fluid was injected into the veins of rabbits at a uniform rapidity and pressure, and the co-efficient of toxicity estimated by the amount of fluid per kilo body weight necessary to produce death in a given time. Control experiments were made with ordinary tap water and distilled water. Under normal conditions the fluid of gastric lavage was found to be but little different in degree of toxicity from tap water, but in cases of hyperacidity the toxic co-efficient was markedly increased and neutralisation of the acid caused a corresponding diminution. One of the most important factors, however, appeared to be motor insufficiency of the stomach, and the degree of toxicity in conditions of gastric ulcer and cancer of the stomach varied according to the motor inactivity of the organ. These results conform well to clinical observations by which a relation between gastric stagnation and general toxæmic symptoms is well established. The highest degree of toxicity was obtained, however, in cases of so-called gastric crises manifested in severe vomiting accompanied by headache, troubles of vision, and other nervous symptoms. The toxicity of the stomach washings during the crisis disappears immediately on cessation of the symptoms, and appears due to some abnormal product of metabolism excreted by the gastric mucous membrane. These results compare well with the favourable effects known to follow gastric lavage, especially in conditions of gastric motor insufficiency, and also in a large number of cases of gastric crises.

The favourable results achieved by Muller and Peiser in the use of human blood-serum and ascitic fluid in cases of acute suppuration have incited M. W. Gilibert to try analogous treatment in cases of ophthalmia neonatorum. A recent number of the *Munch. Med. Woch.* contains a report of eight cases so treated, and the results obtained seem to justify a trial of this treatment. Owing to the facility with which it can be obtained from any slaughter-house, the author employs bovine blood-serum. When kept on ice this serum retains all its physiological properties for at least 24 hours. The method of application of the serum is simple, the child's conjunctivæ being washed out with plenty of serum every two hours. As the result of three weeks of this treatment a cure resulted in most cases without the use of silver nitrate. Even in those cases in which it was necessary to use a silver salt, one or two applications of a 1-per-cent. solution sufficed to complete the cure. The author goes on to say that, in his opinion, the use of serum should not supersede that of silver nitrate, which has stood the test of time; but nevertheless that the new treatment is useful in the intervals between the applications of the silver salt to wash away pus from the conjunctiva. In this way it is possible to reduce the number and the strength of the silver-nitrate applications. Thus, in his opinion, the combined use of serum and silver solutions considerably shortens the duration of the disease.

---

In a recent number of *Le Bulletin Médicale*, Laquerrière discusses the use of electrical treatment in chronic constipation and muco-membranous colitis. In the first place, he insists that electrical treatment must not be considered a substitute for medicinal and dietetic treatment. The effects of electricity are only momentary, and its use cannot be continued for any length of time without danger. There are several methods of applying the current, just as there are several types of constipation. Atony is not the only cause of the loss of proper function, consequently it is not always proper to stimulate the bowel with the interrupted current. The author agrees with Delherm in recognising several types of constipation. The first type he styles that of primitive slight constipation. Here the intestine responds readily to slight stimuli. Treatment consists in looking to the general health of the patient; high frequency, the static electric bath, or the hydro-electric bath are all useful. The next class he calls that of primitive grave constipation. In this type ordinary treatment does not suffice, for it is necessary to distinguish whether the constipation be of the spasmodic or of the atonic variety. In the atonic variety recourse must be had to such treatment as will increase the motive power of the intestine. The interrupted current, the reversed current, static shocks, etc., are all indicated. In the spasmodic variety, however, the continuous current alone is of use, without shocks or reversing of the current. This should be applied at high intensity, 100 milliamperes, or even higher, and large electrodes should be used. If the galvano-faradic current be used, great care should be taken. Other types of constipation come under the heading of symptomatic. If local disease be present, intrarectal applications of the high-frequency current are of use. If the cause of the constipation be a gynæcological one, the high-frequency current is also indicated. Muco-membranous colitis should be treated in the same way as the spasmodic variety of grave constipation.

---

Diabetic dietetics forms the subject of an article in *Le Progrès Medical* by Dr. Morichau-Beauchant, in which he points out the change of view which has taken place in recent years in regard to the influence of the various food-stuffs upon the course of the disease. Briefly put, it is now recognised that glycosuria in diabetics is not only dependent upon the carbohydrates, but also in a large measure upon the proteids ingested. An excess of nitrogenous food increases, and a curtailment diminishes, glycosuria. The limit of tolerance of the carbohydrates is a variable quantity, and is relative to the other kinds of food combined with it. Prout, Bouchardat, and others have from time to time put forward this view, and have shown that an excess of nitrogenous food may cause an increase of glycosuria as well as other symptoms, whereas, on the other hand, by reducing the amount of proteid a much larger quantity of carbohydrates could be tolerated without any increase in the sugar excretion. Kolisch has shown that an excess of proteids may actually cause a comparatively mild form of the disease to take a severe aspect.

---

Quite recently Linossier and Lenoine have by careful observations on two diabetics come to the same conclusions. It is maintained, therefore, that the essential principle of dieting a diabetic consists in a reduction of the total food quantity to a minimum, and it has been shown that metabolic equilibrium can be maintained with diabetics on a much lower minimum of nitrogenous food than with normal individuals. The author of this article agrees with Von Noorden, Kolisch, and others in favouring especially a vegetarian diet, as the vegetable proteid is held to be the least harmful. Another point of practical importance appears to be that carbohydrate food of a certain variety, say potatoes or rice, is much better tolerated when given to the exclusion of all other varieties. To sum up then, the total diet of a diabetic should be reduced as far as possible. The reduction should be made especially in the proteid elements, while as much carbohydrates should be given as can be tolerated without increase in the glycosuria.

---

At a recent meeting of the French Academy of Medicine M. Tessier, of Lyons, has made a most important communication upon the treatment of conditions of nephritis by serum therapy. Following upon the lines of research by Turbure and Renaut with renal extracts and the macerated kidneys of pigs, M. Tessier has ventured to try the effect of renal extracts in cases of nephritis. He used the serum of blood from the renal vein, on the ground that it would contain the renal elements. The serum was first obtained in this way from dogs; but he now uses horses' serum, as it is less toxic. He injects 15 to 20 c.c. of the serum subcutaneously in the abdominal wall of the patients every three or four days. He has tried the method in various types of nephritis, both of an acute and chronic kind, and even in conditions of uræmia. The results have been, according to the author, wonderful. The albuminuria has diminished, uræmia has disappeared, vomiting ceased, and other symptoms have improved or disappeared. It is difficult to form any precise conception of the underlying principles at work in this particular form of opotherapy, although various hypotheses may be formed concerning it. It will be highly interesting to observe whether these results are confirmed by other authorities. In any case it is a new departure in serum-therapeutics which may prove of great value as a remedial measure in kidney disease.

---

The surgical treatment of facial neuralgia formed the subject of an interesting discussion at the recent French Surgical Congress. Resection and avulsion of the peripheral branches of the trigeminal, alcoholic injections, excision of the Gasserian ganglion, division of the main root of the ganglion, resection of the sympathetic, and the results obtained by these various methods all came under review by the various speakers. The experiences of the different surgeons seemed to be remarkably in accord, and it was generally agreed that the minor operations of alcoholic injections or other treatment of the peripheral branches should be given a fair trial in individual cases before the major operation was resorted to. Although with alcoholic injections recurrences are frequent, in the course of six months or more, this form of treatment received a good many advocates on the ground that the operation is trivial, and can easily be repeated without serious inconvenience to the patient. Recurrences, moreover, when they do occur, are generally much diminished in intensity. In cases in which a more radical operation is demanded, the "separation" of the main root of the Gasserian ganglion was held to be preferable to the destruction of the gland itself. Jaboulay, Morestin, and others have found this method equally effectual, and the operation is easier to perform, and is less liable to serious complications and accidents. The evidence in favour of resection of the sympathetic was very various. Some notable successes were recorded, and, on the other hand, many failures have followed the operation. Unfortunately the reason for such variable results is quite unknown. It has at least the advantage over interference with the Gasserian ganglion of being a much less serious operation.

---

Dr. Hertz and others in this country have already carried out important observations upon the functions of digestion in the human subject by administering bismuth salts with the food, and observing the passage of the food through the alimentary canal by means of the $x$-rays, and the results have been reviewed in these columns. Similar observations have been recently carried out by Dr. Kaestle at Munich. He has made more than two hundred experiments on eighty healthy individuals of different ages and both sexes. He finds that the time of duration for the food in the stomach is between two and three and a half hours. At the end of this time the stomach is quite empty. The time appears to be rather longer for women than for men. Peristaltic movements and intermittent relaxation of the pyloric orifice commences almost immediately after the introduction of food. Two to three hours are required for the food to pass through the small intestine. Through the colon the passage of food is very much slower and varies greatly in different individuals. By the same method the author was able to observe the changes from the normal in the process of digestion in morbid conditions, such as pyloric obstruction and atony with dilatation of the stomach. He also made observations on the effect of massage, and the electric current on the stomach, and of purgatives and other drugs. Compared with the stomachs of women, he finds that those of men are wider but not so deep, and occupy a higher position.

---

In the *Archives de Médecine navale* Dr. Coste describes at some length his own symptoms and signs when suffering from an attack of formalin poisoning, the result of handling a large quantity of fish which he was classifying and arranging for the museum of which he is curator. The fish had been preserved in a 5-per cent. solution of formalin. After handling them for some three weeks the author began to suffer from cracked and swollen fingers, intolerable itching, and burning of the skin of the hands, accompanied by a vesicular eruption. He was attacked by a species of hay-fever, accompanied by sneezing and an abundant mucus discharge, which, on drying, stuck to the nasal mucous membrane. His throat was also attacked, giving rise to fits of coughing which caused great pain in the region of the vocal cords, and which only ceased when some of this dried mucous discharge had been coughed up. His general health was at the same time affected; loss of appetite, nausea at the sight of food, general weakness, insomnia, and loss of flesh were all symptoms of the attack of poisoning. The urine smelt of formalin, but was otherwise normal. Bouts of tachycardia were frequent, the pulse reaching 120. There was no increase of temperature, no pain, and no fatigue noticeable after the attacks. These symptoms gradually disappeared in the course of about three weeks. The hands were treated with soothing lotions, and a 1½-per cent. solution of cocaine applied to the throat rapidly cured the cough. No other treatment was adopted.

---

The importance which the use of atoxyl has assumed in the treatment of syphilis, sleeping-sickness, and malignant tumours, has perhaps somewhat obscured the recognition of its usefulness as a tonic when given in smaller doses than is usually the case in the above-mentioned diseases. Dr. M. E. Schacht, in the *Medezinische Klinik*, draws attention to this oversight and recommends the drug in the treatment of anæmia, chlorosis, Graves' disease, tuberculosis, and other wasting diseases. The drug is more efficient than the usual mineral preparations of arsenic and the cacodylates. The author administers the drug by intramuscular injection, using a 10-per cent. solution every second day. He starts by using .02 gram, augmenting each subsequent dose by .02 gram until a dose of .12 gram is reached. Then three doses of .15 gram are given. The injections are next reduced progressively by .02 gram. The total amount of the drug taken is thus about 1½ grams. The author claims that in this way a very much larger quantity of arsenic can be given in a shorter time than is the case when Fowler's solution is being taken as a tonic, and moreover patients can be so treated who are unable to tolerate the ordinary arsenic preparations.

